# A Treatise on the Anatomy and Physiology of the Teeth, &c. &c. Their Diseases and Treatment; with Practical Observations on Artificial Teeth, and Rules for Their Construction

**Published:** 1841-12

**Authors:** 


### Bibliographical Notices.

*A Treatise on the Anatomy and Physiology of the Teeth, &c. &c. their diseases and treatment; with practical observations on Artificial Teeth, and rules for their construction.* By David Wemyss Jobson, M. R. C. S., &c. 2d edition, London, 1835, pp. 270, 8vo.

The above work, although published six years ago, is but little known in the United States. It was our intention to have noticed it at a much earlier period, but the amount of other matter which we have had upon our hands, has compelled us to restrict our bibliographical department to such narrow limits, that we have been unable to do so. We shall for the future, however, endeavour to be more prompt in presenting to our readers, notices of, at least, all new publications relating to the teeth.

The object of the author of the treatise under review, seems to have been the instruction of the medical, rather than the dental practitioner. His opinions, for the most part, accord with those of Hunter, Fox and Bell. The doctrine of the non-vascularity of dental bone, as maintained by Hunter, he does not adhere to, but in other respects he differs from him but little. As to the literary character of his work, it is highly creditable, and besides, he seems to be familiar with the writings of most English authors on dental science. His strictures upon the use of the "anodyne cement," and "mineeral succedanea," that have been so much puffed by a certain class of dental empirics in Great Britain, are no less just than true, and were medical gentlemen generally, aware of the injury that results from this species of quackery, they would be less ready, than many seem to be, to aid, by commending to public confidence, every one who applies to them for recommendatory testimonials. To determine upon the merits of any description of dental practice, it is necessary to be acquainted with it, and to know its effects and the competency of the practitioner.

As there is nothing peculiar from other authors in Mr. Jobson's views concerning the anatomy, physiology and dentition of the teeth, we shall pass, without further remark, that portion of his work which is devoted to these subjects. They, as well as all the subjects embraced within his treatise, are treated on very briefly, and yet, sufficiently at length, to convey to the reader a good general knowledge of them. The brevity of his work then, may be regarded as a recommendation rather than as an objection, and the more especially as it is designed more for the general than the dental practitioner, whose other studies and professional duties, leave him little leisure for the cultivation of a branch of the profession, whose duties he is seldom, if ever, called upon to exercise. It is as necessary, however, that he should have a general knowledge of the physiological and pathological relationships that exist between the dental apparatus and other parts of our physical being, as it is for the surgeon dentist to be acquainted with the structure and diseases of the other parts of the body, and Mr. Jobson's work furnishes this information.

But while we in the main approve of the plan of the work, and coincide with the author in most of the views which he advances, we cannot assent to all. He has adopted some opinions so obviously erroneous, that we cannot allow them to pass unnoticed.

In treating on irregularities of the teeth, he says, "that a crowded condition of these organs will take place, 'notwithstanding every care on the part of the dentist,' in those cases where their dimensions exceeds those of the jaws. Now, this is a proposition to which we should be very reluctant to subscribe; and, we do not hesitate to allege, that, by timely and judicious care, any disposition to irregularity resulting from this cause, may be so overcome as to secure symmetry and regularity to the arrangement of the denture. The means that have, in hundreds of instances, been successfully employed for the prevention of the evil he condemns, and from the manner in which he speaks of them, we are disposed to think that he has had but little experience in this department of practice. The only successful plan that has ever been adopted, in cases of this kind, consists in the extraction of a tooth on each side of the jaw. This, if done at the proper time, will always enable the dental practitioner to procure for his patient a well arranged set of teeth, but to this practice, though the object be accomplished by it, Mr. Jobson objects. He says, "that the want of the teeth is always evident, and occasions a contraction of the jaws, which materially impairs the symmetry of the face." It may lessen the length of the circle of the alveolar ridge, we admit, but instead of "impairing the symmetry of the face," it has an opposite effect. Though two of the teeth, one on each side, be wanting, their absence will not, if the rest be correctly arranged, be observable.

The practice of filing the teeth with a view to remedy an irregularity, as recommended by some French and English writers, we conceive to be exceedingly objectionable. Thousands of teeth have been ruined by it, and not because the operation necessarily injures the organs, but because when performed for this purpose, the filed surfaces are soon brought in contact and afford a lodgment for morbid secretions and extraneous matter, which by being retained, exerts a destructive action both upon the enamel and dental bone; or, in other words, it causes them to decay. It is therefore, important that, when the teeth are filed for the removal of caries, that the operation should be performed in such a way as to prevent the organs from coming together, else it, instead of proving beneficial, will become a source of injury. It is an operation that is very often called for, and when correctly and judiciously performed, is of incalculable value.

Mr. Jobson, however, admits that there are cases in which extraction is called for, but he does not mention the teeth that are to be removed, but we infer from what he says, that the mal-placed organs are the ones which he would take away, and if we are correct in this, we must object to the practice. We will quote what he says upon the subject. "There are instances," says he, "indeed, as in the case of the upper canines, when they perforate high up on the anterior surface of the jaw, in which early extraction will be necessary." Now the proper practice in cases of this kind, (except the subjects be passed the eighteenth year of their age, when, in many persons, the deformity can only be removed by the extraction of the projecting cuspidati,) is to extract the first or second bicuspis on each side.

When the upper incisors come out behind the circle of the other teeth, the means which he employs to bring them forward consists of a gold bar passed round in front of the arch, with blocks of ivory, as recommended by Mr. Fox, attached to each end so as to interpose between the teeth and prevent them from coming together. The gold bar, he says, is an invention of Mr. Hunter. If Mr. Jobson had been conversant with French authors, he would have discovered that Mr. Hunter was not entitled to the credit of the invention of this instrument. A drawing of it may be seen in the "Recherches du Dentiste," by Bourdet, vol. 2, between the 18th and 19th pages, and this work was published 1757, fourteen years before the publication of the first part of Mr. Hunter's treatise on the teeth, and twenty-one years before the publication of that part in which he treats on irregularity. But even this instrument will not, in those cases where any of the upper incisors have come out so far back as to fall behind or on the inside of the lower, enable us to bring them forward without the interposition of blocks of ivory, or some other substance, between the back teeth. Gold caps, for the last fifteen or twenty years have, been substituted. They are more convenient, and are in many other respects decidedly preferable. M. Delabarre, a French dentist and author of considerable celebrity, recommends the placing of a gold grate, made of wire, over two of the lower molares, but there are many cases, in which this would not keep the teeth sufficiently apart.

It sometimes happens, that the lower incisors shuts over the upper, and to prevent which, Mr. Jobson employs a kind of guttered plate, similar to the one described by M. Duval, and M. Catalan. This is a most excellent method, the lower teeth can, with very great facility, after the extraction of one on each side, be carried back by the aid of this fixture, and latterly we have been induced to employ it for moving upper front ones that stood within the the circle, forward. There are many cases in which we prefer it to any other means that have been recommended.

In treating on caries of the teeth, he advocates the doctrine that the disease is the result of inflammation of the bony tissue of the organs, but uses no arguments that have not before been advanced in its support. He quotes the explanation, given by Mr. Bell, of the cause, as fully expressive of his own views upon the subject. But, of the importance of cleanliness of the teeth, he seems to be fully aware, and very properly recommends it; but at the same time, he very justly condemns the use of acid dentifrices, so much employed by "empirical practitioners," with a view to impart to the teeth a temporary whiteness. The injury produced upon the teeth by this description of quack nostrums is incalculable.

The absurdity of the doctrine, advocated by Mr. Jobson, of the decay of the teeth, has been so frequently shown, that we do not deem it necessary to say any thing more upon the subject. The more rational and philosophical explanation, that the malady is the result of the action of solvent agents, we are gratified to believe, is fast gaining ground.

Among the remedies recommended by the author for tooth-ache, we shall notice but one, and that is the nitrate of silver. Of the caustics, this he conceives to be the best. The nitrate of silver will sometimes succeed in destroying the lining membrane and ganglion of a tooth, but it is a very uncertain remedy, and its application is always followed by a great augmentation of pain, and the irritation produced by it not unfrequently extends to the alveolar periosteum and gums. The only caustic that has been used with complete success, is arsenic; and the profession are greatly indebted to Dr. Spooner, of Montreal, and his brother, Dr. S. Spooner, of New York, who were the first to recommend it in this country. We were somewhat sceptical at first concerning its effects, but having employed it in some two or three hundred cases, we no longer feel any hesitation about recommending it is as an infallible remedy. Different practitioners are in the habit of applying it differently. We have ourself used it combined with various articles, but the preparation which we prefer, both for convenience and as giving less pain to the patient, is the following: arsenic and acetate of morphine, each one grain, mix and divide into twenty parts, upon a particle of raw cotton about the size of a pin's head, or a little larger, previously moistened in water, we absorb one of these powders, and after drying the cavity in the tooth, apply it to the exposed ganglion. This done, we fill the cavity with yellow wax. The application is rarely followed with much pain, and when it is, it seldom continues for more than from three to five hours, and when made directly to the ganglion, or pulp, within the cavity of the tooth, a second application is never requisite. We have been in the habit of applying it one day, and plugging the tooth the next.

Exostosis, necrosis, fractures, and dislocations of the teeth, salivary calculus, and the diseases of the gums and alveolar process are treated on in the fifth, sixth, and seventh chapters of the second part of Mr. Jobson's work. After which a chapter on the diseases of the maxillary bones follows, and then another on neuralgia of the face. Not observing any thing peculiar in what the author's says concerning these maladies, we pass them without comment.

Surgical and mechanical dentistry constitute the subjects of the third and fourth parts of the treatise, and it is to be regretted, that the author has not described more at length the various manipulations pertaining to them. No one can obtain sufficient knowledge from the brief description which he has given of the operations belonging to dental surgery, to enable him to cleanse, file, plug or insert a tooth in a proper or scientific manner. Operations upon the teeth, to be valuable, must be well performed, and no one can perform them well who is not thoroughly acquainted with all their minutia. So far as Mr. Jobson has made known his views of practice, we have little fault to find with them. We, however, object to the names by which he designates some operations; for example, that of plugging the teeth, he calls stuffing; filling, stoppmg or plugging; we should have greatly preferred, but as we are not disposed to be over fastidious about names, we shall proceed, without further comment upon them, to notice two or three more passages, and conclude our remarks upon his work.

With the directions which he lays down, for the preparation of a cavity in a tooth, previously to filling, we are pleased.

He says, "In excavating the cavity, although it is necessary to clean away the whole of the diseased structure, and it is desirable to make the interior of the opening as equal as possible—yet it should be the study of the operator to avoid removing any of the sound part of the tooth, with the view of rendering the space wider beneath than it is at the surface."

Many practitioners, though not almost every one as Mr. Jobson says, recommend that the cavity should be made larger at the bottom than it is at the opening. It is by far more difficult to fill a cavity thus formed, than when it is all the way of a size, and when it is considerably larger at the bottom than it is at the opening, it cannot be well filled. This is one of most difficult operations in dental surgery, and requires more judgment and skill in its performance than any other. Very few, comparatively, have arrived to a high degree of excellence in this department of practice.

The author's remarks upon the materials most proper to be employed for filling teeth, are deserving of especial notice. His views upon this will be found to accord with those of the most scientific and skilful practitioners of the day.

"Of all the materials" he observes, "that have been employed for this purpose, gold leaf, the most ancient, is still incomparably the best, as it is not only the purest of all the metals, but is possessed of the highly valuable property of undergoing no change in the mouth, where almost every other metallic substance is affected. Platina, the more recently discovered metal, and inferior only to gold, it is true, forms an exception to this remark; but it is rarely to be found in a state of purity, and is even then possessed of much less ductility than gold is."

To the filing of the teeth, an operation, which, in the treatment of caries on their lateral surfaces, is of inestimable value, he gives but about one and a half pages. That he should have passed with so few remarks, an operation that is of so much importance, is, to us, a matter of surprise. The directions which he gives upon the subject are comprised in about half a dozen lines, and they are too general to be serviceable as a guide to the practitioner. The following is pretty nearly all he says concerning the operation.

"In the first stages of the disease," (caries upon the lateral surfaces of the teeth,) "the operation requires but little ingenuity, as it is merely necessary to make a small space, by passing a smooth file between the teeth. But when the caries is more extensive, considerable care is requisite to effect the removal of the diseased part without materially altering the outward aspect of the tooth."

Now what are we to understand, "by passing a smooth file between the teeth." If we are to place no other construction upon the words than their literal meaning, we unhesitatingly pronounce the practice a bad one, for by that simple operation no protection whatever will be afforded to the teeth against decay. The disease would be favoured by it rather than otherwise. Though the caries be removed, the teeth will soon come together, and thus a lodgment, as we have before stated will be afforded for extraneous and impure matters, and the disease be again speedily produced. When teeth are to be filed, the operation should be so performed as to prevent the approximation of the organs.

Tying teeth that have become loosened in their sockets, from the use of mercury, or any other cause, with a ligature, with a view to prevent them from moving, is a practice, which, we are sorry to find, he recommends. It is unscientific, and highly reprehensible. Although they may for a time be kept somewhat steadier, a cure of the disease in the gums and alveoli can never be accomplished, while teeth which require this kind of artificial support, are permitted to remain in them. If then we would prevent an extension of the evil, we should advise their removal at once.

The instruments employed by Mr. Jobson, for the extraction of teeth, are the key, forceps, punch, and elevator. In most cases he gives the preference to the forceps, but says there are cases in which a resort to the key must be had, and repeats what Mr. Bell says about those who are loudest to condemn this instrument, that they have a secret recourse to it. We are not surprised that he should think so, nor would any one be, who would take the trouble to look at the drawings of the forceps which he has given. They are such clumsily and badly contrived things, that really we should hardly know how to proceed to effect the evulsion of a tooth with them. The difficulty in extracting teeth with forceps, has been the want of such as were properly constructed and adapted to the purpose, but that is now obviated, we have them now with which any tooth can be removed, and with less injury to the alveoli and gums, than is inflicted with the best constructed key instrument now in use, and with less physical force. If there are any who are yet sceptical upon this subject, we say to them, if they will supply themselves with forceps suitable for the purpose, and use them exclusively for six months, our word for it, they will never go back to the key instrument. We have given both a fair trial, and therefore speak from experience on the subject.

The origination of the barbarous operation of transplanting teeth from the mouth of one person to that of another, Mr. Jobson, ascribes to Mr. Hunter. It is surprising that most English writers on the teeth attribute the invention of this operation to Mr. Hunter, when it was performed in France about two hundred years before he was born.

Since the time of the publication of the work under review, the improvements in the manner of inserting artificial teeth have been so great, that we do not think it necessary to notice that part of it which is devoted to this branch of the art. The directions given upon this subject, so far as they go, would have answerd at that time, and some of them are applicable even now. In this department of the profession, Mr. Jobson was well informed.

In conclusion, we can only say, that though some of the doctrines maintained by the author, do not accord with established facts, and sound philosophy, his treatise as a whole is creditable, and if not intended for the surgeon dentist, is certainly well worthy of a place in his library.—*Balt. Ed.*

